# Evaluation of two Massive Open Online Courses (MOOCs) in genomic variant interpretation for the NHS workforce

**DOI:** 10.1186/s12909-023-04406-x

**Published:** 2023-07-28

**Authors:** Beth Coad, Katherine Joekes, Alicja Rudnicka, Amy Frost, Mark Robert Openshaw, Katrina Tatton-Brown, Katie Snape

**Affiliations:** 1 St George’s University of London, London, UK; 2 National Genomics Education, NHS England, London, UK; 3 Institute of Cancer and Genomics, University of Birmingham, Birmingham, UK

**Keywords:** Genomics education, MOOCs, Online learning, Genomic variants, Cancer genomics

## Abstract

**Background:**

The implementation of the National Genomic Medicine Service in the UK has increased patient access to germline genomic testing. Increased testing leads to more genetic diagnoses but does result in the identification of genomic variants of uncertain significance (VUS). The rigorous process of interpreting these variants requires multi-disciplinary, highly trained healthcare professionals (HCPs). To meet this training need, we designed two Massive Open Online Courses (MOOCs) for HCPs involved in germline genomic testing pathways: Fundamental Principles (FP) and Inherited Cancer Susceptibility (ICS).

**Methods:**

An evaluation cohort of HCPs involved in genomic testing were recruited, with additional data also available from anonymous self-registered learners to both MOOCs. Pre- and post-course surveys and in-course quizzes were used to assess learner satisfaction, confidence and knowledge gained in variant interpretation. In addition, granular feedback was collected on the complexity of the MOOCs to iteratively improve the resources.

**Results:**

A cohort of 92 genomics HCPs, including clinical scientists, and non-genomics clinicians (clinicians working in specialties outside of genomics) participated in the evaluation cohort. Between baseline and follow-up, total confidence scores improved by 38% (15.2/40.0) (95% confidence interval [CI] 12.4–18.0) for the FP MOOC and 54% (18.9/34.9) (95%CI 15.5–22.5) for the ICS MOOC (*p* < 0.0001 for both). Of those who completed the knowledge assessment through six summative variant classification quizzes (V1–6), a mean of 79% of respondents classified the variants such that correct clinical management would be undertaken (FP: V1 (73/90) 81% Likely Pathogenic/Pathogenic [LP/P]; V2 (55/78) 70% VUS; V3 (59/75) 79% LP/P; V4 (62/72) 86% LP/LP. ICS: V5 (66/91) 73% VUS; V6 (76/88) 86% LP/P). A non-statistically significant higher attrition rate was seen amongst the non-genomics workforce when compared to genomics specialists for both courses. More participants from the non-genomics workforce rated the material as “Too Complex” (FP *n* = 2/7 [29%], ICS *n* = 1/5 [20%]) when compared to the specialist genomics workforce (FP *n* = 1/43 [2%], ICS *n* = 0/35 [0%]).

**Conclusions:**

After completing one or both MOOCs, self-reported confidence in genomic variant interpretation significantly increased, and most respondents could correctly classify variants such that appropriate clinical management would be instigated. Genomics HCPs reported higher satisfaction with the level of content than the non-genomics clinicians. The MOOCs provided foundational knowledge and improved learner confidence, but should be adapted for different workforces to maximise the benefit for clinicians working in specialties outside of genetics.

**Supplementary Information:**

The online version contains supplementary material available at 10.1186/s12909-023-04406-x.

## Background

The use of genomic sequencing in clinical practice continues to expand, with many more patients now being offered germline genomic testing as part of their standard NHS care [1–4]. Increased germline genomic testing increases the potential for early diagnosis, targeted treatment and cancer prevention options through the identification of germline genomic variants related to disease predisposition. In order to realise this potential, the challenge of providing robust genomic variant interpretation and the clinical translation of genomic data into accurate risk predictions with evidence-based management, must be overcome.

International guidelines have been introduced by the American College of Medical Genetics and Genomics (ACMG) to assist with evidence-based variant interpretation [5]. Further guidance has also been provided by specialist groups such as the Association for Clinical Genomic Science (ACGS) [6], Cancer Variant Interpretation Group UK (CanVIG-UK) [7] and ClinGen [8]. These detailed guidelines apply a myriad of scientific evidence to variant interpretation and rely on a high level of specialist knowledge.

### Training needs

While detailed published national and international guidance is the first step in ensuring equitable standardised high-quality variant interpretation, specialist workforce training in the application of this guidance is also required. The specialist genomics workforce in the NHS includes clinical scientists, clinical geneticists and genetic counsellors. Thus far in the UK, variant interpretation training has been delivered in silos, focusing on pockets of the specialist genomics workforce [9–12]. However, intensive face-to-face training is expensive and time consuming and can only target a small number of individuals at any one time.

In addition, ordering genomic testing, interpreting the results and adapting clinical management on the basis of these results is no longer solely the responsibility of specialist clinical genomics services. Genomic testing may also be requested by clinicians working in specialties outside of genetics from secondary and tertiary care and is often referred to as mainstream testing [2, 4]. A growth of this model will allow more individuals to access testing, and result in return of results to non-genomic specialists. To appropriately manage a patient following a genomic test result, a non-genomics specialist will need to have an understanding of the variant classification system, the immediate management of variants of uncertain significance, and may also participate in multi-disciplinary meetings where variant interpretation is discussed, for example a Genomics Tumour Advisory Board (GTAB).

In the oncology setting, Tutika et al. highlighted that oncology trainees and consultants lacked confidence in interpreting germline genomic variants of uncertain significance (VUS) and communicating the results to patients [13]. These training needs also apply to other areas of medicine in which genomic testing is common, with the majority of paediatricians and other non-cancer clinicians being unable to appropriately answer variant quizzes in different studies [14, 15].

### Challenges of delivering variant interpretation education in the NHS

There are various reasons for this continuing training need. Genomics, and variant interpretation specifically, is a continuously evolving area of medicine. The Can-VIG UK group alone released five new gene-specific guidelines between March 2020 and January 2022 [7], and the ACMG is expected to release a significant update to their 2015 recommendations [5] imminently. This constant movement creates shifting sands for workforce training needs. Frequent evolution combined with the complex skill requirements for variant interpretation mean that the specialist genomics workforce need detailed training on the immovable fundamentals early in their career, with real-time access to flexible and responsive training on the latest updates in the field.

A further challenge is presented by the time-poor NHS workforce who require flexible, accessible and wide-reaching options for continuing education. In the wake of the COVID-19 pandemic and an increasing familiarity with virtual conferencing platforms, distance learning options such as Massive Open Online Courses (MOOCs) would meet this need.

### MOOCs

Massive Open Online Courses, including many covering medical education, are openly accessible online courses that are available to learners globally [16, 17], with notable utilisation seen during the COVID-19 pandemic [18–20].

While genomics MOOCs exist [21–23], there is a lack of detailed content focusing on variant interpretation in the NHS. In order to meet this training need and address the challenges above, we designed and implemented two MOOCs in a collaborative approach between the CanGene-CarVar programme [24], the Genomics Education Programme (GEP) of Health Education England (HEE) [25], and St George’s University of London, in Partnership with the MOOC platform FutureLearn [21].

The first MOOC, ‘Interpreting Genomic Variation: Fundamental Principles’ (FP), focuses on the foundational knowledge required for genomic variant interpretation in rare disease, including explorations of the ACMG [5] and ACGS [6] guidelines for variant interpretation. Building upon the FP MOOC, the second MOOC, ‘Interpreting Genomic Variation: Inherited Cancer Susceptibility’ (ICS), provides more in-depth training on interpreting variation in cancer susceptibility genes grounded in the CanVIG-UK guidance [7].

To assess these educational resources, we implemented an evaluation course run using a pilot group of learners. The development of these MOOCs and the evaluation design meet the criteria suggested in the Reporting Item Standards for Education and its Evaluation in Genomics (RISE2 Genomics) [26].

This evaluation aimed to consider:

- Objective evidence of improved knowledge after undertaking the MOOC(s)
- Subjective user-reported feedback on confidence in managing VUS after undertaking the MOOC(s)
- Detailed learner feedback across workforce roles to adapt and improve the MOOCs for both the genomics and non-genomics specialist workforces

## Methods

### Course design and delivery

The MOOCs were designed using an iterative process [22], with content creation led by a working group of subject matter experts including clinical genetics and cancer genetics consultants, genetics specialist registrars (SpRs), clinical scientists and a genetic counsellor. A curriculum map was used to plan the content for each MOOC [27], with content focused on active learning pedagogies.

The FP MOOC Is a 3-week course, with approximately 4 h of study time per week, and the ICS MOOC Is a 2-week course, with 3 h of study time per week [See Supplementary Table 1, Additional File 1]. *Recruitment to the evaluation cohort.*

Free online access to the MOOCs was available from 10^th^ January 2022 on the FutureLearn platform. No advertising was undertaken by the course team with respect to the MOOC launch but it was possible for learners to sign up online and access MOOC materials from this point.

A voluntary response sampling method was used to invite participants from a range of NHS clinical workforces involved in genomic testing to participate in the first runs of the MOOCs as part of an evaluation cohort. The genomics specialist workforce was then subdivided to ensure participants spanned various experience levels, from trainees to consultants. Participants were initially invited through a range of professional email groups including the Genomic Medicine Service Alliances (GMSAs), UK Cancer Genetics Group (UKCGG), Association for Genetic Nurses and Counsellors (AGNC), CanVIG-UK and Clinical Genetics Trainees group. A snowball sampling method was used to recruit non-genomics specialists as these individuals were harder to reach with the initial mailout. Recruitment to the evaluation cohort required commitment to complete the pre- and post- course surveys, alongside MOOC completion. In return, participants were offered upgraded access to the MOOC materials.

**Fig. 1:**
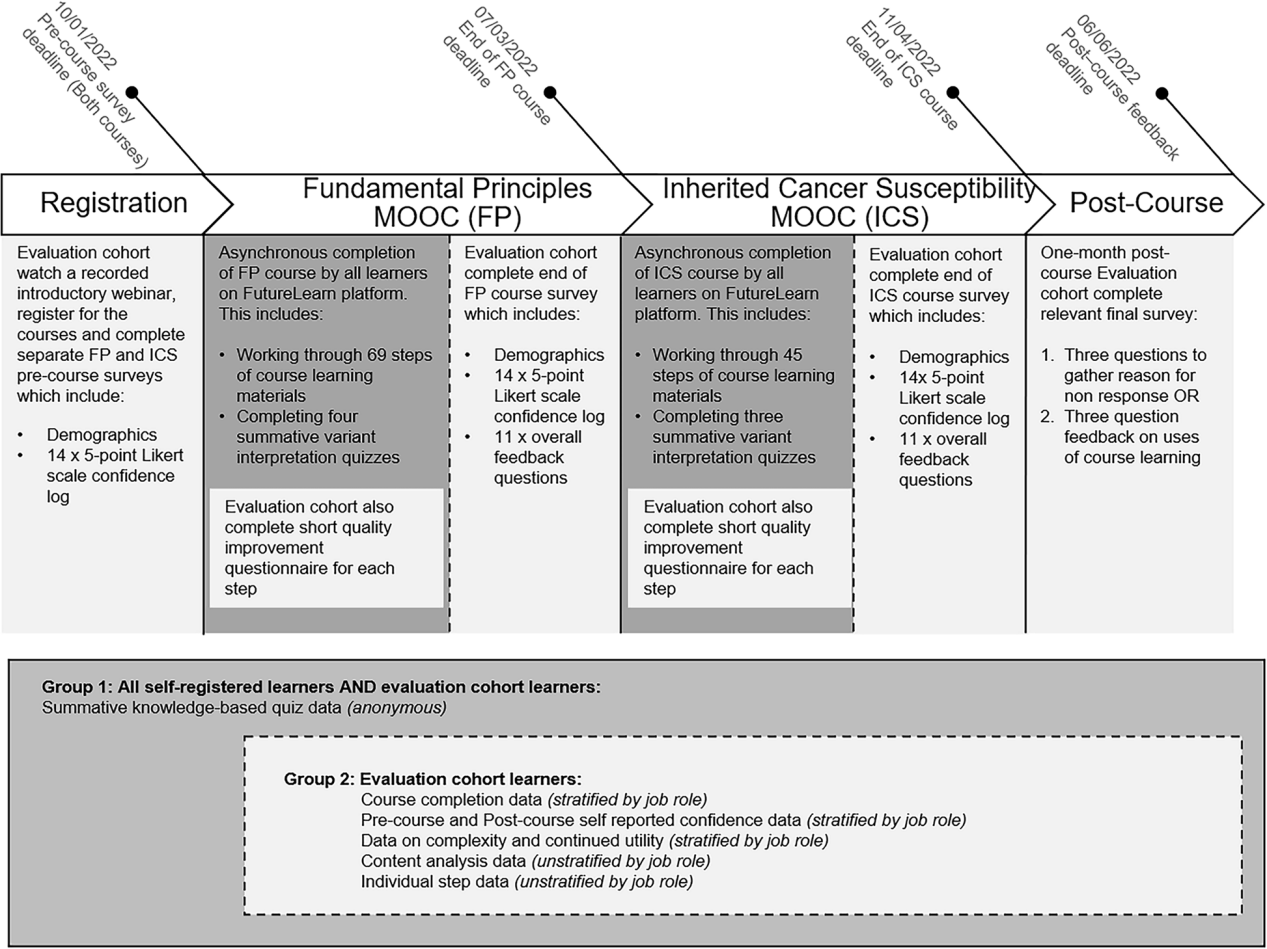
Timeline and design of evaluation methods with outline of learner groups who contributed evaluation data

This resulted in two groups of learners; 1) Learners who self-registered and completed the course external to the evaluation cohort 2) Learners who registered as part of the evaluation cohort, gave demographic details and committed to providing evaluation data (See Fig. 1).

Data that was obtained from in-MOOC variant interpretation quizzes included all learners from both group 1 and group 2. It was not possible to subdivide these learners further from the internal MOOC quiz data. Data from the pre- and post- course surveys and content analysis included only those in the evaluation cohort for whom demographic data was available.

### Evaluation methods

A longitudinal evaluation with varied methods was used to gather data prior to, during and after each MOOC. The outputs included subjective self-reported confidence scores and course feedback, in addition to objective quizzes to assess knowledge. An overview of the various stages of evaluation can be seen in Fig. 1.

The courses were openly accessible to all learners (including evaluation cohort participants) through the online FutureLearn platform from launch on 10^th^ January 2022. This platform provides anonymous feedback data on course completion rates, participation in discussion boards and quiz scores; this includes the results of summative quizzes in the final section of each course, which ask learners to work through the process of classifying a series of genomic variants.

To gather workforce-specific feedback on learner confidence, satisfaction, perceived difficulty, and continued utility of the course, external surveys were sent to all evaluation cohort participants. These surveys requested demographic data alongside a 5-point Likert scale for self-reported assessment of confidence [See Supplementary Table 2, Additional File 1].

### Data analysis

#### Comparison of pre-course and post-course self-reported confidence data (Group 2: Evaluation Cohort)

Fundamental principles and ICS pre- and post-course surveys contained a series of confidence questions with Likert responses from ‘strongly agree’ to ‘strongly disagree’. These responses were converted to scores from 1 to 5 (possible range of scores from 14 to 70). Non-parametric statistics (Wilcoxon matched-pairs signed-rank test for equality of matched pairs of observations) were used to compare total scores between baseline and follow-up for both surveys. In addition, questions were grouped into the following domains: ‘communication’, ‘team working’ and ‘knowledge/skills’ to assess whether changes in scores between baseline and follow-up differed across domains [See Supplementary Table 2, Additional File 1]. All analyses were performed using STATA 16 (StataCorp LLC USA).

#### Summative knowledge-based quiz scores (Group 1: All learners)

All learners who completed any of the variant interpretation quizzes up to April 2022 were included in this analysis. These data were anonymous and included learners taking part in the MOOCs who were not part of the evaluation cohort.

Responses from six variant interpretation quizzes (V1–6) were downloaded from the FutureLearn platform (FP = 4, ICS = 2). Variant 7 (V7) was excluded as the data presented deliberately led to an erroneous classification if benignity data were not considered. The number of learners correctly classifying the variant and whether the classification of the variant would have led to a correct clinical management plan were analysed and compared across both courses.

#### Feedback on complexity (Group 2: Evaluation cohort)

Overall complexity, rated by participants in the end of course surveys, was given a numerical value of 1 (too simple), 2 (about right) or 3 (too complex). Responses from the genomics and non-genomics workforce were compared.

#### Other data (Group 2: Evaluation cohort)

Non-response rate and continued utility of the course resources were analysed from additional questionnaires sent to participants 1 month after the ICS course deadline.

Step-by-step feedback was iteratively reviewed and used to make real-time minor adjustments such as the correction of errors, e.g., spelling errors and broken web links. This data is not presented here but will be used to improve subsequent versions of the course.

#### Content analysis (Group 2: Evaluation cohort)

The range of qualitative data gathered from the post-course survey was analysed separately for each MOOC and themes were suggested [28]. Two authors (KJ and BC) each coded a section of the free text. The codes from each author were then combined to further refine categories and themes. Deductive themes informed by the wider literature and grounded inductive coding were used to allow novel themes to emerge [29].

## Results

### Participant characteristics (Group 2: Evaluation Cohort)

Overall, 133 HCPs responded to invitation emails to participate in the evaluation cohort. Some non-cancer specialists did not wish to complete the ICS course; 84% (*n* = 112/133) registered to take both the FP and ICS courses. The cohort who completed the evaluation comprised genomics specialist HCPs with varying experience (including trainee and registered scientists/genetic counsellors, and clinical/cancer genetics SpRs and consultants), as well as non-genomics specialists across oncology, haematology, paediatrics and cardiology.

From this group of volunteers, 69% (*n* = 92/133) of participants went on to complete the pre-course survey for the FP course, with 79% (*n* = 73/92) of these participants going on to complete the pre-course survey for the ICS course [See Supplementary Table 3, Additional File 1].

**Fig. 2:**
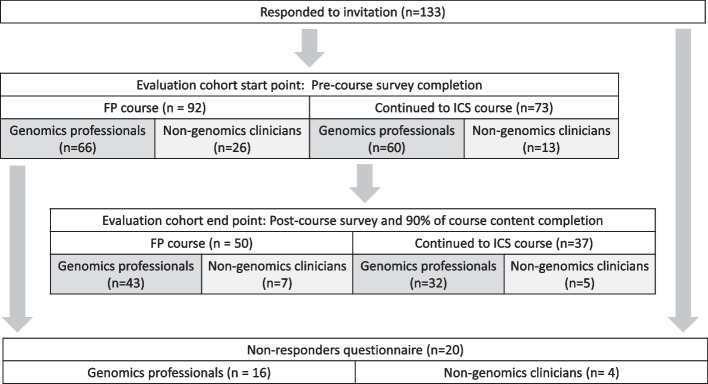
Attrition rates of learners in the evaluation cohort comparing genomics and non-genomic professionals

Attrition in completion of the course material and corresponding end of course surveys was seen throughout the evaluation, as seen in Fig. 2. This attrition was most significant amongst non-genomics clinicians. For the FP course, despite 26 non-genomics clinicians completing the pre-course survey, only 27% (*n* = 7/26) went on to complete the full evaluation (≥ 90% of the course material and the post-course survey), compared to 65% (*n* = 43/66) of genomics HCPs. This difference was seen to a lesser extent with the ICS course, in which 39% (*n* = 5/13) of non-genomics clinicians who submitted the pre-course survey went on to complete the evaluation, compared to 53% (*n* = 32/60) of genomics HCPs. This difference was not statistically significant but shows a trend towards higher attrition amongst the non-genomics workforce.

A survey was circulated to participants who responded to the initial invitation and were not able to complete 90% of the courses within the evaluation period (*n* = 83) to ascertain the reasons for this (Response rate = 24%, 20/83). 94% (15/16) of genomics professionals and 100% (4/4) of non-genomics clinicians stated their reason for non-completion was lack of time in their role for this training.

### Evaluation of learner confidence (Group 2: Evaluation cohort)

Between baseline and follow-up, total confidence scores improved by 15.2 out of a possible 29.9 points (95% confidence interval [CI] 12.4–18.0) for the FP MOOC and 18.9 out of a possible 35.1 points (95%CI 15.5–22.5) for the ICS MOOC (*p* < 0.0001 for both), with improvements across different domains outlined in Table 1. This equates to a total percentage increase in confidence of 38% for the FP and 54% for the ICS.

**Table 1:** Increase in average self-reported confidence scores from baseline to follow-up across three domains of variant interpretation practice (in all instances *p* < 0.0001)

**Domain**	Increase in confidence scores (95% confidence interval)	Total Pre course confidence	Total Post course confidence	Percentage increase in confidence	Increase in confidence scores (95% confidence interval)	Total Pre course confidence	Total Post course confidence	Percentage increase in confidence
	**FP**				**ICS**			
**Communication**	4.4 (3.2–5.5)	16.7	21.1	26.3%	6.1 (4.8–7.5)	14.8	20.9	41.2%
**Team working**	7.0 (5.6–8.4)	16.0	23.0	43.8%	7.9 (6.4–9.5)	14.3	22.2	55.2%
**Knowledge/skills**	3.8 (3.2–4.5)	7.3	11.1	52.1%	4.9 (4.0–5.8)	5.8	10.7	84.5%
**Total**	**15.2**	40.0	55.2	**38.0%**	**18.9**	34.9	53.8	**54.2%**

*FP* Fundamental Principles, *ICS* Inherited Cancer Susceptibility

**Fig. 3:**
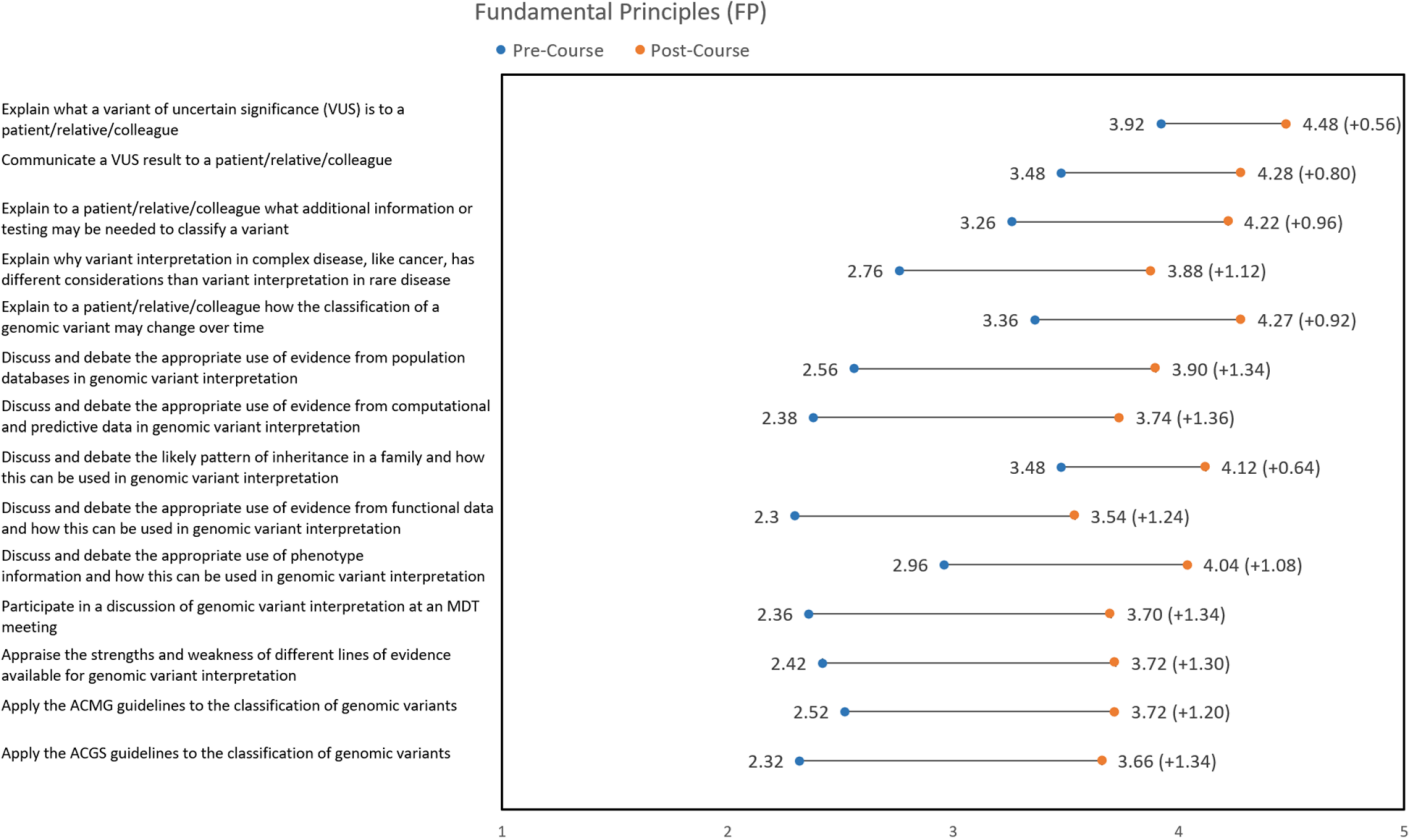
Increase in average learner confidence scores (1 = strongly disagree, 2 = disagree, 3 = neither agree nor disagree, 4 = agree, 5 = strongly agree) for each question in Fundamental Principles (FP)

**Fig. 4:**
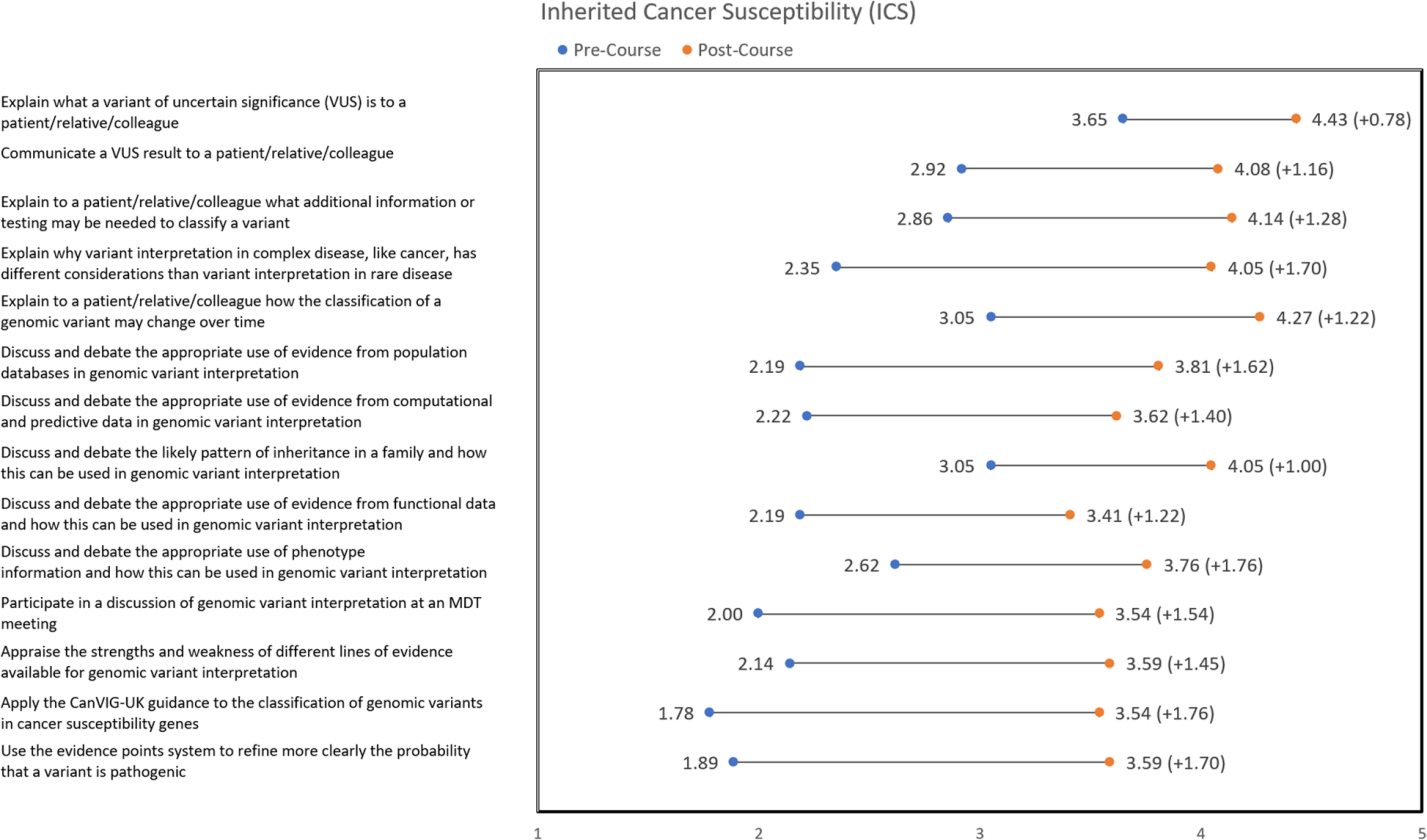
Increase in average learner confidence scores (1 = strongly disagree, 2 = disagree, 3 = neither agree nor disagree, 4 = agree, 5 = strongly agree) for each question in Inherited Cancer Susceptibility (ICS)

Improvements in confidence were seen across all questions for FP (see Fig. 3) and ICS (see Fig. 4). *Knowledge assessments (Group 1: All learners).*

All learners on the course had the opportunity to undertake the variant interpretation quizzes (FP = 557 and ICS = 357 total course enrolments). The majority of learners in the FP course correctly classified three out of four variants to the five-point ACMG scoring system (Table 2). Clinical management of a variant however is binary – Class 4 and 5 variants are treated as disease associated and clinically actionable, and Class 1, 2 and 3 variants are treated as non-clinically actionable for the purposes of management of the condition in question. The *WDR26* (NM_025160.6) c.490C > T p.(Arg164Ter) variant was correctly classified as Class 5 (Pathogenic) by only 32% (*n* = 24/75) of learners. However, the majority of those who did not classify it as Class 5 classified it as Class 4 (Likely Pathogenic) and therefore the correct clinical management would be followed. Considering clinical actionability, the number of respondents correctly classifying the variant was: V1 81% Likely Pathogenic/Pathogenic (LP/P); V2 70% VUS; V3 79% LP/P; V4 86% LP/LP.

**Table 2:** Breakdown of variant classifications given by learners in course exercises

Course	Fundamental Principles (FP)																			
Variant	*V1: FOXG1* (NM_005249.4) c.695A > G p.(Asn232Ser)					*V2: NANS* (NM_018946.4) c.452G > A p.(Arg151His)					*V3: WDR26* (NM_025160.6) c.490C > T p.(Arg164Ter)					*V4: REEP1* (NM_022912.2) c.471del p.(Thr158fs)				
No. of learners	90					78					75					72				
Variant classification	B	LB	VUS	**LP**	*P*	B	LB	**VUS**	LP	*P*	B	LB	VUS	LP^a^	***P***	B	LB	VUS	**LP**	*P*
No. of learners	2.2% (2)	3.3% (3)	13.3% (12)	**71.1% (64)**	10.0% (9)	2.6% (2)	11.5% (9)	**70.5% (55)**	12.8% (10)	2.6% (2)	2.7% (2)	6.7% (5)	12.0% (9)	46.7% (35)	**32.0% (24)**	0.0% (0)	5.6% (4)	8.3% (6)	**65.3% (47)**	20.8% (15)
Course	Inherited Cancer Susceptibility (ICS)																			
Variant	*V5: CSG123* (NM_000045.6) c.786A > G p.(Thr262Ala)^b^					*V6: MLH1* (NM_000249.3) c.122A > G p.(Asp41Gly)					*V7: BRCA2* (NM_000059.4) c.8351G > A p.(Arg2784Gln)									
No. of learners	91					88					83									
Variant classification	B	LB	**VUS**	LP	*P*	B	LB	VUS	LP	***P***	B		LB		VUS		**LP **^**c**^		*P*	
No. of learners	3.3% (3)	8.8% (8)	**72.5% (66)**	13.2% (12)	2.2% (2)	2.3% (2)	4.5% (4)	6.8% (6)	18.2% (16)	**68.2% (60)**	6.0% (5)		3.6% (3)		24.1% (20)		**63.9% (53)**		2.4% (2)	

*B* Benign, *LB* Likely Benign, *VUS* Variant of Uncertain Significance, *LP* Likely Pathogenic, *P* Pathogenic Correct classifications are in bold

These data were anonymous and included learners taking part in the MOOCs who were not part of the evaluation cohort

^a^ Likely Pathogenic would be the correct classification under ACMG criteria alone, however, Pathogenic is correct as per ACGS adaptions

^b^ Variant fabricated as a pedagogical device to scaffold learning

^c^ With the information provided in the exercise, this was the appropriate classification, however, with broader data, VUS would be the correct final classification

Most learners in the ICS course correctly classified all three variants (Table 2). The course deliberately withheld data for V7, *BRCA2* (NM_000059.4) c.8351G > A p.(Arg2784Gln), when asking learners to classify the variant to highlight the importance of considering data for benignity as well as pathogenicity in classification, and therefore this variant was not considered in this analysis. Considering clinical actionability, the number of respondents correctly classifying the variant was: V5 73% VUS; V6 86% LP/P.

Across both MOOCs, a mean of 77.5% of learners classified the six variants such that the correct clinical action plan would be followed.

As this data was anonymous, it was not possible to differentiate between learners from different professional backgrounds, including those not part of the research cohort who completed the evaluation.

### Learner satisfaction and perceived difficulty (Group 2: Evaluation Cohort)

In this section, we present the feedback from all evaluation participants who completed the post-course survey. In the ICS course, this includes three participants who did not complete the ICS pre-course survey and therefore were not included in the confidence analysis above.

The majority of participants completing feedback on satisfaction agreed that the courses were engaging (FP *n* = 50/50 [100%]; ICS *n* = 39/40 [98%]) and that they enjoyed the MOOC style of learning (FP *n* = 48/50 [96%]; ICS *n* = 36/40 [90%]). All participants (100%) agreed that the learning was relevant to their professional development and would recommend the courses to a colleague.

A minority of participants did not agree that they had the opportunity to interact with other learners (FP *n* = 10/50 [20%]; ICS *n* = 9/40 [22.5%]).

Most participants agreed that the overall course difficulty was at ‘about the right level’ (FP *n* = 47/50 [94%]; ICS *n* = 38/40 [95%]). A greater proportion of non-genomics clinicians (FP *n* = 2/7 [29%]; ICS *n* = 1/5 [20%]) rated either or both of the courses’ content as ‘too complex’ compared to the specialist genomics HCPs (FP *n* = 1/43 [2%]; ICS *n* = 0/35 [0%]). This was not statistically significant.

### Content analysis (Group 2: Evaluation Cohort)

For the final part of the post-course survey, free text questions were answered by 32 individuals who completed both courses. A further 18 participants provided free text feedback on the FP course alone, and 8 participants completed this for the ICS course only. Full details of themes are available in supplementary materials [Supplementary Table 4, Additional File 1].

Across both courses, many learners (FP *n* = 24, ICS *n* = 24) commonly stated that they enjoyed the quizzes, which took them step-by-step through the interpretation of a variant identified in a clinical case. Other enjoyable aspects across both courses were the videos which included patients’ stories (FP *n* = 14, ICS *n* = 10). While not drawn out as a specific theme, as applicable to most education, videos also included learning from experts in the field, which was appreciated by some participants.

*‘…the videos from experts were very informative and it was great learning about a specific topic from individuals with significant contributions to the area. I also found the patient experiences very important as they serve to remind us of the true purpose behind the science.’ (Trainee Genetic Counsellor)*

Overall feedback was positive, with free text answers suggesting that the course was comprehensive; many felt that no information was missing (FP *n* = 38, ICS *n* = 29) and many suggested that no improvements were required (FP *n* = 15, ICS *n* = 13).

Areas for improvement and low enjoyment were also similar across both courses, with themes arising around text-heavy information presentation (FP *n* = 15, ICS *n* = 6) and the need for more visual content (FP *n* = 5, ICS *n* = 8). Issues around the online nature of the course were also highlighted, with learners wanting more interaction and feedback. Some learners also highlighted the complexity of the content and felt that it may have been too difficult for their role, suggesting a potential improvement to simplify material for non-genomics clinicians (FP *n* = 5, ICS *n* = 3).

*‘There was a lot of text to read through which was understandably full of jargon so difficult to read. I think the course was perhaps too in depth for me…’ (Oncology Trainee)*

### Continued utility (Group 2: Evaluation Cohort)

The free text analysis suggested that many learners planned to return to the materials after completing the course. When surveyed 2 months after course completion, 24/30 (80%) of genomics professionals and 7/7 (100%) non- genomics clinicians had accessed the course at least once for continued learning. In this short time frame 18/30 (60%) of genomics professionals, and 5/7 (71%) non-genomics clinicians had reviewed the content at least once to support a work task.

*‘I can’t thank you enough for this course as this has been an eye opener for me. My understanding of variant interpretation has definitely improved and I will definitely look back to it for reference.’ (Pre-registration Clinical Scientist)*

## Discussion

Both courses received positive feedback from participants and learners showed a significant self-assessed improvement in confidence in germline variant interpretation after completing each course. The majority of responders were able to correctly classify both rare disease and cancer germline variants through summative quizzes, with the exception of an instance in which recent guidelines supersede the initial variant classification criteria outlined by the ACMG [5], highlighting the need to emphasize the changing nature of guidance in this area.

This study is the first to consider differences in outcomes between genomics and non-genomics HCPs completing the same MOOCs. While feedback was generally positive across both groups, with increased learner confidence reported, non-genomics clinicians found many steps too complex and showed higher rates of attrition. This may primarily be due to less in-depth genomics knowledge and fewer opportunities to practice variant interpretation for those ordering mainstream genomic tests [30–32]. This suggests that tailored training for this limited workforce may be required.

### Limitations

While most learners were able to correctly classify genomic variants having completed the courses, this study did not have data on learner demographics to aid further analysis, and did not consider learner knowledge prior to MOOC completion. In addition, the time limitations of this study meant it was not able to assess if this knowledge was successfully applied to practice. There remains a lack of standardisation amongst assessment methods in healthcare MOOCs, with many evaluations not reporting this data [13, 17, 33, 34]. Challenges to robust assessment in these MOOCs included the varied audience of learners due to the open access format, difficulty authentically assessing variant interpretation competence in an online setting and the time limitations of the study.

While attrition was seen amongst all learners, rates were lower than those often reported for online learning [17, 33, 35]. The voluntary response sampling method used in this study could have resulted in a biased sample of participants, with those in the genomics workforce who felt most in need of further training being over-represented. It is also likely that the snowball sampling approach attracted non-genomics specialists with an existing keen interest in genomics. Therefore, the low attrition rates potentially reflect a high prior motivation to learn amongst participants, as seen with other medical education MOOCs [36, 37]. In addition, the sample size of this study was small, in particular for non-genomics healthcare professionals. This means that the results may not be representative of all HCPs involved in genomic testing in the UK.

The anonymisation of some data, such as the variant interpretation quizzes, did not enable learner confidence scores to be compared with individual knowledge assessments or allow stratification to differentiate between the different professional groups.

The number of learners taking part in the courses may have been limited as they had not yet been widely advertised, this was to allow feedback from the evaluation cohort to be used to improved content before wider dissemination amongst all HCPs who order genomic testing in the UK. This smaller cohort may have resulted in lower learner participation in forums and discussions. This may mean fewer participants benefited from social learning, which is often highlighted as a core benefit of MOOC pedagogy [16, 17, 38], and was highlighted by our learners requests for more interaction in their free text feedback.

### Future work

Participant free text feedback highlighted a desire for more interaction and the challenges of the asynchronous nature of the MOOCs. A possible alternative approach would be a mentor-led model, which has shown success in another genomic MOOC [22], or a blended approach with some face-to-face tutorials. This may improve learner experience and provide opportunity for further application of knowledge learnt. This blended model of learning has been found to be non-inferior to traditional face-to-face teaching [39, 40], and has seen better performance when compared with ‘MOOC-only’ learners [41].

Further work is needed to develop resources that are tailored to the needs of non-genomics clinicians, considering the complexity and real-world application of the knowledge required. Additional research is necessary to fully understand what these different educational needs are. This research should also consider the long-term impacts of this education on practice. In this evolving specialty, best practice guidelines around variant interpretation are rapidly changing. Consequently, frequent updates to this MOOC will be required to keep the content relevant, with a blended learning style providing opportunities for learners to apply the most up-to-date guidance. Granular step-by-step learner feedback from this evaluation can also be used to improve learner experience along with updates to the content.

## Conclusions

Self-reported confidence in germline genomic variant interpretation amongst HCPs significantly increased after completing one or both MOOCs (*p* < 0.0001) The use of real-world clinical cases and worked examples successfully allowed learners to engage with the MOOCs’ active learning led approach. Approximately 80% of respondents could correctly classify variants such that appropriate clinical management would be instigated. Genomics HCPs reported higher satisfaction regarding the level of content than the non-genomics workforce. The MOOCs provided foundational knowledge, which was valued by learners and improved learner confidence, but should be adapted for different workforces to maximise the benefit for those working outside of clinical genomics. Further studies are needed to consider how best to assess subjective knowledge improvements and skills application in variant interpretation, and to evaluate the long-term implications of this educational intervention on HCPs’ practice.

## Supplementary Information

**Additional file 1: Supplementary Materials Table 1.** Curriculum map.** Supplementary Materials Table 2.** Survey confidence questions in 3 domains each with Likert responses from ‘strongly agree’ to ‘strongly disagree’. These responses were converted to scores from 1 to 5 (possible range of scores from 14 to 70).** Supplementary Materials Table 3.** Job role of participants completing pre-course survey.** Supplementary Materials Table 4.** Content categories of free text answers.

## Data Availability

The datasets used and/or analysed during the current study are available from the corresponding author on reasonable request.

## Abbreviations

ACMG: American College of Medical Genetics and Genomics
ACGS: Association for Clinical Genomic Science
AGNC: The Association for Genetic Nurses and Counsellors
B: Benign
CanVIG-UK: Cancer Variant Interpretation Group UK
CI: Confidence Interval
FP: Fundamental Principles
GEP: Genomics Education Programme
GMSAs: Genomic Medicine Service Alliances
HCPs: Healthcare professionals
HEE: Health Education England
ICS: Inherited Cancer Susceptibility
LB: Likely Benign
LP: Likely Pathogenic
MOOC: Massive Open Online Course
P: Pathogenic
SpRs: Specialist Registrars
UKCGG: The UK Cancer Genetics Group
VUS: Variant(s) of Uncertain Significance

## References

[1] England HE (2019). The Topol Review: Preparing the healthcare workforce to deliver the digital future.

[2] Snape K, Wedderburn S, Barwell J (2019). The new genomic medicine service and implications for patients. Clin Med.

[3] Josephs KS, Berner A, George A, Scott RH, Firth HV, Tatton-Brown K (2019). Genomics: the power, potential and pitfalls of the new technologies and how they are transforming healthcare. Clin Med (Lond).

[4] NHS. NHS Long Term Plan. 2019. Available from: www.longtermplan.nhs.uk.

[5] Richards S, Aziz N, Bale S, Bick D, Das S, Gastier-Foster J (2015). Standards and guidelines for the interpretation of sequence variants: a joint consensus recommendation of the American college of medical genetics and genomics and the association for molecular pathology. Genet Med.

[6] Ellard S, Baple E, Callaway A, Berry I, Forrester N, Turnbull C, et al. ACGS best practice guidelines for variant classification in rare disease 2020. 2020. Available from: https://www.acgs.uk.com/media/10793/uk_practice_guidelines_for_variant_classification_2018_v10.pdf.

[7] Garrett A, Durkie M, Callaway A, Burghel GJ, Robinson R, Drummond J (2021). Combining evidence for and against pathogenicity for variants in cancer susceptibility genes: CanVIG-UK consensus recommendations. J Med Genet.

[8] ClinGen. Variant Pathogenicity Curation [Available from: https://clinicalgenome.org/curation-activities/variant-pathogenicity/.

[9] Sciences NSoH. Curriculum Library - Scientist Training Programme: Health Education England; 2022 [Available from: https://curriculumlibrary.nshcs.org.uk/stp/.

[10] Programme GE. Bioinformatics, Interpretation, Statistics & Data Quality Assurance in Genomics: Health Education England; 2022 [Available from: https://www.genomicseducation.hee.nhs.uk/education/taught-courses/bioinformatics-interpretation-statistics-data-quality-assurance-in-genomics/.

[11] London SGUo. Genomic Medicine PGCert PgDip MSc London: St Georges University; 2022 [Available from: https://www.sgul.ac.uk/study/courses/genomic-medicine#structure.

[12] Science WC. Clinical Genomics: Fundamentals of Variant Interpretation in Clinical Practice: Wellcome Connecting Science 2020 [Available from: https://coursesandconferences.wellcomeconnectingscience.org/event/clinical-genomics-fundamentals-of-variant-interpretation-in-clinical-practice-20200129/.

[13] Tutika RK, Benett J, Abraham J, Snape K, Tatton-Brown K, Kemp Z, et al. Mainstreaming of genomics in oncology: a nationwide survey of genomics training needs of UK oncologists. Personal Communication. In Press.10.7861/clinmed.2022-037236697012

[14] Menke C, Nagaraj CB, Dawson B, He H, Tawde S, Wakefield EG (2021). Understanding and interpretation of a variant of uncertain significance (VUS) genetic test result by pediatric providers who do not specialize in genetics. J Genet Couns.

[15] Macklin SK, Jackson JL, Atwal PS, Hines SL (2019). Physician interpretation of variants of uncertain significance. Fam Cancer.

[16] Liyanagunawardena TR, Williams SA (2014). Massive open online courses on health and medicine: review. J Med Internet Res.

[17] Longhini J, De Colle B, Rossettini G, Palese A (2021). What knowledge is available on massive open online courses in nursing and academic healthcare sciences education? A rapid review. Nurse Educ Today.

[18] Bhattacharya S, Singh A, Hossain MM (2020). Health system strengthening through Massive Open Online Courses (MOOCs) during the COVID-19 pandemic: An analysis from the available evidence. J Educ Health Promot.

[19] Ismail II, Abdelkarim A, Al-Hashel JY. Physicians’ attitude towards webinars and online education amid COVID-19 pandemic: When less is more. PLoS ONE. 2021;16(4): e0250241. 10.1371/journal.pone.0250241 PMC8051773 33861799

[20] Shah D. By the Numbers: MOOCs During the Pandemic: Class central; 2020 [Available from: https://www.classcentral.com/report/mooc-stats-pandemic/.

[21] FutureLearn. FutureLearn platform 2022 [Available from: https://www.futurelearn.com/.

[22] Bishop M, Miller E, McPherson A, Simpson S, Sutherland S, Seller A (2019). Genomic education at scale: the benefits of massive open online courses for the healthcare workforce. Front Genet.

[23] Coursera. Coursera platform [Available from: https://www.coursera.org/.

[24] CanGen-CanVar. The CanGene-CanVar Programme 2022 [Available from: www.cangene-canvaruk.org.

[25] HEE. Health Education England Genomics Education Programme 2022 [Available from: https://www.genomicseducation.hee.nhs.uk/.

[26] Nisselle A, Janinski M, Martyn M, McClaren B, Kaunein N, Maguire J (2021). Ensuring best practice in genomics education and evaluation: reporting item standards for education and its evaluation in genomics (<em>RISE2 Genomics</em>). Genet Med.

[27] Pickering JD, Henningsohn L, DeRuiter MC, de Jong PGM, Reinders MEJ (2017). Twelve tips for developing and delivering a massive open online course in medical education. Med Teach.

[28] Glaser B, Strauss A (1967). The discovery of grounded theory: Strategies for qualitative research.

[29] Saldaña J. The coding manual for qualitative researchers. 2nd edition ed. Arizona: SAGE; 2013.

[30] Hallowell N, Wright S, Stirling D, Gourley C, Young O, Porteous M. Moving into the mainstream: healthcare professionals’ views of implementing treatment focussed genetic testing in breast cancer care. Fam Cancer. 2019;18(3):293–301. 10.1007/s10689-019-00122-y PMC6560008 30689103

[31] Al Bakir I, Sebepos-Rogers GM, Burton H, Monahan KJ (2019). Mainstreaming of genomic medicine in gastroenterology, present and future: a nationwide survey of UK gastroenterology trainees. BMJ Open.

[32] Eccles BK, Copson E, Maishman T, Abraham JE, Eccles DM (2015). Understanding of BRCA VUS genetic results by breast cancer specialists. BMC Cancer.

[33] Aldahdouh A, Osório A (2016). Planning to design MOOC? Think first!. Online J Distance Educ e-Learning.

[34] Rowe M, Osadnik CR, Pritchard S, Maloney S (2019). These may not be the courses you are seeking: a systematic review of open online courses in health professions education. BMC Med Educ.

[35] Evans DP, Luffy SM, Parisi S, del Rio C (2017). The development of a massive open online course during the 2014–15 Ebola virus disease epidemic. Ann Epidemiol.

[36] Pham T, Beloncle F, Piquilloud L, Ehrmann S, Roux D, Mekontso-Dessap A (2021). Assessment of a massive open online course (MOOC) incorporating interactive simulation videos on residents’ knowledge retention regarding mechanical ventilation. BMC Med Educ.

[37] Magaña-Valladares L, Rosas-Magallanes C, Montoya-Rodríguez A, Calvillo-Jacobo G, Alpuche-Arande CM, García-Saisó S (2018). A MOOC as an immediate strategy to train health personnel in the cholera outbreak in Mexico. BMC Med Educ.

[38] Guest C, Wainwright P, Herbert M, Smith IM (2021). Driving quality improvement with a massive open online course (MOOC). BMJ Open Qual.

[39] Cao W, Hu L, Li X, Chen C, Zhang Q, Cao S (2021). Massive open online courses-based blended versus face-to-face classroom teaching methods for fundamental nursing course. Medicine (Baltimore).

[40] Bowen WG, Chingos MM, Lack KA, Nygren TI (2014). Interactive learning online at public universities: evidence from a six-campus randomized trial. J Policy Anal Manage.

[41] Jia M, Gong D, Luo J, Zhao J, Zheng J, Li K (2019). Who can benefit more from massive open online courses? A prospective cohort study. Nurse Educ Today.

