# Supplementary material for: Evaluation of two Massive Open Online Courses (MOOCs) in genomic variant interpretation for the NHS workforce
Source: BMC Med Educ. 2023 Jul 28;23:540. doi: 10.1186/s12909-023-04406-x (PMC10386229; doi:10.1186/s12909-023-04406-x)
Supplement: Supplementary file 1 — Additional file 1: Supplementary Materials Table 1. Curriculum map. Supplementary Materials Table 2. Survey confidence questions in 3 domains each with Likert responses from ‘strongly agree’ to ‘strongly disagree’. These responses were converted to scores from 1 to 5 (possible range of scores from 14 to 70). Supplementary Materials Table 3. Job role of participants completing pre-course survey. Supplementary Materials Table 4. Content categories of free text answers. [file 12909_2023_4406_MOESM1_ESM.doc]

Supplementary Materials Table 1: Curriculum map

| Interpreting genomic variation: Fundamental Principles (FP) | | |
| --- | --- | --- |
| Week one | Week two | Week three |
| Introduction | Computational and predictive data | ACMG & ACGS guidelines |
| Normal genetic variation | Functional data | VUS Multi-disciplinary meetings |
| Changing technologies | Reputable databases |  |
| Variant Classification | Phenotyping and literature searches | Genomic variant interpretation in practice |
| Population databases | Ethics of genomic variant interpretation |  |

| Interpreting genomic variation: Inherited Cancer Susceptibility (ICS) | |
| --- | --- |
| Week one | Week two |
| Introduction | Functional data |
| Differences in Cancer Susceptibility Genes | Allelic data |
| Adapting the ACMG framework | Reputable sources |
| Population data | Other data |
|  | Other consideration |
| Computational and predictive data | In practice |
|  | The evolving Landscape |

Supplementary Materials Table 2: Survey confidence questions in 3 domains each with Likert responses from ‘strongly agree’ to ‘strongly disagree’. These responses were converted to scores from 1 to 5 (possible range of scores from 14 to 70).

| Fundamental Principles (FP) | |
| --- | --- |
| Domain | Question |
| Communication | *Explain what a variant of uncertain significance (VUS) is to a patient/relative/colleague* |
|  | *Communicate a VUS result to a patient/relative/colleague* |
|  | *Explain to a patient/relative/colleague what additional information or testing may be needed to classify a variant* |
|  | *Explain why variant interpretation in complex disease, like cancer, has different considerations than variant interpretation in rare disease* |
|  | *Explain to a patient/relative/colleague how the classification of a genomic variant may change over time* |
| Team-Working | *Discuss and debate the appropriate use of evidence from population databases in genomic variant interpretation* |
|  | *Discuss and debate the appropriate use of evidence from computational and predictive data in genomic variant interpretation* |
|  | *Discuss and debate the likely pattern of inheritance in a family and how this can be used in genomic variant interpretation* |
|  | *Discuss and debate the appropriate use of evidence from functional data and how this can be used in genomic variant interpretation* |
|  | *Discuss and debate the appropriate use of phenotype information and how this can be used in genomic variant interpretation* |
|  | *Participate in a discussion of genomic variant interpretation at an MDT meeting* |
| Knowledge/Skills | *Appraise the strengths and weakness of different lines of evidence available for genomic variant interpretation* |
|  | *Apply the ACMG guidelines to the classification of genomic variants* |
|  | *Apply the ACGS guidelines to the classification of genomic variants* |
| Inherited Cancer Susceptibility (ICS) | |
| Domain | *Question* |
| Communication | *Explain what a variant of uncertain significance (VUS) is to a patient/relative/colleague* |
|  | *Explain why variant interpretation in complex disease, like cancer, has different considerations than variant interpretation in rare disease* |
|  | *Communicate a cancer susceptibility genes (CSG) VUS result to a patient/relative/colleague* |
|  | *Explain to a patient/relative/colleague what additional information or testing may be needed to classify a variant* |
|  | *Explain to a patient/relative/colleague how the classification of a genomic variant may change over time* |
| Team-Working | *Discuss and debate the appropriate use of evidence from population databases in variant interpretation in CSGs* |
|  | *Discuss and debate the appropriate use of evidence from computational and predictive data in variant interpretation in CSGs* |
|  | *Discuss and debate the likely pattern of inheritance in a family and how this can be used in CSG variant interpretation* |
|  | *Discuss and debate the appropriate use of evidence from functional data and how this can be used in CSG variant interpretation* |
|  | *Discuss and debate the appropriate use of phenotype information and how this can be used in CSG variant interpretation* |
|  | *Participate in a discussion of CSG variant interpretation at an MDT meeting* |
| Knowledge/Skills | *Appraise the strengths and weakness of different lines of evidence available for genomic variant interpretation in CSGs* |
|  | *Apply the CanVIG-UK guidance to the classification of genomic variants in cancer susceptibility genes* |
|  | *Use the evidence points system to refine more clearly the probability that a variant is pathogenic* |

Supplementary Materials Table 3: Job role of participants completing pre-course survey

| Job role | No. of participants |
| --- | --- |
| Genomics HCPs | |
| Clinical Scientists: Trainee (STP) | 2 |
| Clinical Scientists: Pre-registration | 6 |
| Clinical Scientist: Registered | 8 |
| Clinical/Cancer Genetics: Specialist Registrar (SpR) | 17 |
| Clinical/Cancer Genetics: Consultant | 11 |
| Genetic Counsellor: STP Trainee/MSc Student | 8 |
| Genetic Counsellor: Pre-registration | 4 |
| Genetic Counsellor: Registered | 10 |
| Non-Genomics HCPs | |
| Oncology: Trainee | 5 |
| Oncology: Consultant | 2 |
| Breast Associate Specialist | 1 |
| Consultant Paediatric Oncologist | 1 |
| Paediatrics: Trainee | 2 |
| Paediatrics: Consultant | 3 |
| Cardiology: Consultant | 4 |
| Haematologist | 3 |
| Pathologist | 3 |
| Neurology: Consultant | 1 |
| Gastroenterologist | 1 |

Supplementary Materials Table 4: Content categories of free text answers

| **Fundamental Principles** | | | | | | | | | | | |
| --- | --- | --- | --- | --- | --- | --- | --- | --- | --- | --- | --- |
| What did you enjoy most about the course? | | What did you enjoy least about the course? | | Was there any information you felt was missing from the course? | | Was there any part of the course you found particularly easy? | | Was there any part of the course you found particularly difficult? | | How could we improve the course? | |
| Quizzes (worked cases) | 24 | Information presentation (e.g. Text heavy) | 15 | No | 38 | No | 27 | No | 12 | None suggested | 15 |
| Videos | 14 | Complexity | 9 | Further guidance on external websites | 4 | Intro | 8 | Databases | 10 | Additional content | 6 |
| Content | 12 | None | 9 | Genomic basics | 2 | Clinical | 2 | Applying guidelines | 8 | More visual content | 5 |
| Clinical relevance | 11 | Online asynchronous (lack of feedback) | 5 | Splicing content | 1 | Videos | 1 | Segregation | 5 | More stratification for different clinical roles | 5 |
| Structure | 7 | Time commitment | 4 | More quizzes | 1 | Cases | 1 | Quizzes | 5 | More quizzes | 5 |
| Resources | 6 | External links | 2 | Gain of function variants content | 1 | MDT | 1 | Phenotyping | 1 | Technical errors | 3 |
| Variety | 5 | Databases | 2 | Benignity | 1 | Ethics | 1 | functional evidence | 1 | Structure | 3 |
| Interactive | 2 | Long quizzes | 1 | Clinician input | 1 | All | 1 |  |  | More clinical relevance | 2 |
| Own pace | 1 | Lack of interaction | 1 | More content (general) | 1 | ACMG framework | 1 |  |  | Lack of interaction | 2 |
|  |  |  |  | More segregation content | 1 | Quizzes | 1 |  |  | Reduce difficulty | 2 |
|  |  |  |  |  |  |  |  |  |  | Widen access | 1 |
|  |  |  |  |  |  |  |  |  |  | Screenshots | 1 |
|  |  |  |  |  |  |  |  |  |  | Worked examples | 1 |
|  |  |  |  |  |  |  |  |  |  |  |  |
|  |  |  |  |  |  |  |  |  |  |  |  |
|  |  |  |  |  |  |  |  |  |  |  |  |
|  |  |  |  |  |  |  |  |  |  |  |  |
|  |  |  |  |  |  |  |  |  |  |  |  |
|  |  |  |  |  |  |  |  |  |  |  |  |
|  |  |  |  |  |  |  |  |  |  |  |  |
|  |  |  |  |  |  |  |  |  |  |  |  |
|  |  |  |  |  |  |  |  |  |  |  |  |
| **Inherited cancer susceptibility** | | | | | | | | | | | |
| What did you enjoy most about the course? | | What did you enjoy least about the course? | | Was there any information you felt was missing from the course? | | Was there any part of the course you found particularly easy? | | Was there any part of the course you found particularly difficult? | | How could we improve the course? | |
| Quizzes (worked cases) | 24 | None | 15 | None | 29 | No | 30 | No | 14 | None suggested | 13 |
| Videos | 10 | Complexity | 5 | Genomic basics | 3 | Clinical | 5 | Collating evidence | 4 | More visual content | 8 |
| Continued learning | 5 | Information presentation (e.g. Text heavy) | 6 | Lacking practical information | 2 | Intro | 2 | Evidence points system | 3 | More feedback | 3 |
| Structure | 5 | Time commitment | 3 | More clashing classifications content | 1 | Phenotype | 1 | Databases | 3 | Worked examples | 3 |
| Clinical relevance | 4 | Lack of interaction | 3 | More tumour testing detail | 1 | Case scoring | 1 | External websites | 2 | More stratification for different clinical roles | 3 |
| Content | 4 | External links | 2 | Other cancer genes | 1 |  |  | Population calculations | 2 | More patient stories | 2 |
| Resources | 2 | Online asynchronous (lack of feedback) | 2 | Resource section | 1 |  |  | Gene specific guidance | 1 | Resource section | 2 |
| Online discussion | 2 | Length | 2 | Further guidance on external websites | 1 |  |  | Phenotyping | 1 | Lack of interaction | 2 |
|  |  | Technical issues | 2 |  |  |  |  | Clashing classification | 1 | More quizzes | 2 |
|  |  | Lacking practical information | 1 |  |  |  |  | Functional data | 1 | More on functional studies | 1 |
|  |  | Repetitive | 1 |  |  |  |  | All | 1 | Update | 1 |
|  |  |  |  |  |  |  |  | Guidelines | 1 | Clashing classification | 1 |
|  |  |  |  |  |  |  |  |  |  | More audio content | 1 |
|  |  |  |  |  |  |  |  |  |  | Links to specific part of guidance | 1 |
|  |  |  |  |  |  |  |  |  |  | More clinical relevance | 1 |
